# Cellular dynamics during regeneration of the flatworm *Monocelis* sp. (Proseriata, Platyhelminthes)

**DOI:** 10.1186/2041-9139-5-37

**Published:** 2014-10-23

**Authors:** Johannes Girstmair, Raimund Schnegg, Maximilian J Telford, Bernhard Egger

**Affiliations:** Research Unit of Evolutionary Developmental Biology, Institute of Zoology, University of Innsbruck, Technikerstrasse 25, 6020 Innsbruck, Austria; Department of Genetics, Evolution and Environment, University College London, Darwin Building, Gower Street, WC1E 6BT London, UK; Research Unit of Ecotoxicology, Institute of Zoology, University of Innsbruck, Technikerstrasse 25, 6020 Innsbruck, Austria

**Keywords:** blastema, flatworm, planarian, proliferation, proseriate, regeneration, turbellarian

## Abstract

**Background:**

Proseriates (Proseriata, Platyhelminthes) are free-living, mostly marine, flatworms measuring at most a few millimetres. In common with many flatworms, they are known to be capable of regeneration; however, few studies have been done on the details of regeneration in proseriates, and none cover cellular dynamics. We have tested the regeneration capacity of the proseriate *Monocelis* sp. by pre-pharyngeal amputation and provide the first comprehensive picture of the F-actin musculature, serotonergic nervous system and proliferating cells (S-phase in pulse and pulse-chase experiments and mitoses) in control animals and in regenerates.

**Results:**

F-actin staining revealed a strong body wall, pharynx and dorsoventral musculature, while labelling of the serotonergic nervous system showed an orthogonal pattern and a well developed subepidermal plexus. Proliferating cells were distributed in two broad lateral bands along the anteroposterior axis and their anterior extension was delimited by the brain. No proliferating cells were detected in the pharynx or epidermis.

*Monocelis* sp. was able to regenerate the pharynx and adhesive organs at the tip of the tail plate within 2 or 3 days of amputation, and genital organs within 8 to 10 days. Posterior pieces were not able to regenerate a head.

The posterior regeneration blastema was found to be a centre of cell proliferation, whereas within the pharynx primordium, little or no proliferation was detected. The pharynx regenerated outside of the blastema and was largely, but not solely formed by cells that were proliferating at the time of amputation.

**Conclusions:**

Our findings suggest that proliferating cells or their offspring migrated to the place of organ differentiation and then stopped proliferating at that site. This mode of rebuilding organs resembles the mode of regeneration of the genital organs in another flatworm, *Macrostomum lignano*. Pharynx regeneration resembles embryonic development in *Monocelis fusca* and hints at the vertically directed pharynx being plesiomorphic in proseriates.

Proliferation within the regeneration blastema has been detected in anterior and posterior blastemas of other flatworms, but is notably missing in triclads. The phylogenetic relationships of the flatworms studied indicate that proliferation within the blastema is the plesiomorphic condition in Platyhelminthes.

**Electronic supplementary material:**

The online version of this article (doi:10.1186/2041-9139-5-37) contains supplementary material, which is available to authorized users.

## Background

Proseriates represents a species-rich (over 400 species have been described), widespread, and predominantly marine group of hermaphroditic free-living flatworms (Platyhelminthes) [1]. It is split into the Unguiphora (without a statocyst) and the Lithophora (possessing a statocyst) [2], the latter including the common genus *Monocelis*, which is characterized by paired eyes that have merged, forming a single eyespot directly anterior to the statocyst [3].

Flatworms have been famous for centuries, owing to their regenerative powers. Indeed, they were among the first animals in which regeneration was studied. Pallas, in 1774, was the first to notice that a small piece of a triclad (planarian) head is capable of regenerating a complete organism [4] and Dalyell concluded in 1814 after his experiments with *Polycelis nigra* that this species may ‘almost be called immortal under the edge of the knife’ [5]. Since then, most of the effort in understanding the regeneration capacity of free-living flatworms has been concentrated on triclads. There it has been shown that a population of pluripotent stem cells called neoblasts, capable of differentiating into any other cell type and the only proliferating cells in the organisms, forms the basis of regeneration [6]. Apart from triclads, other taxa of the free-living flatworms are known to regenerate, although to different extents [7]. Unfortunately, many of these flatworm groups are understudied and lack the application of more contemporary methods, such as visualization of their stem cell system by 5-bromo-2′-deoxyuridine (BrdU) labelling, which could provide comparative insights into the regeneration processes of free-living flatworms. This lack of information holds true for flatworms of the order Proseriata, in which the most recent overview of their powers of regeneration was published almost 50 years ago [8].

In the earliest record of regeneration in proseriates, von Graff [9] mentions having cut the proseriate *Monocelis fusca* into two or three pieces, where the pieces containing the head continued their fast movement, whereas middle and posterior pieces only resumed moving after three to five days.

Another regeneration study was carried out on the freshwater species *Otomesostoma auditivum* (Proseriata, Lithophora). Steinböck [10] found that, owing to their compact shape, many animals died soon after cutting them crossways in two halves. Anterior pieces including the head with brain and eyes and a portion of the gut, but without the pharynx, typically died a few hours after amputation. Posterior halves without the head but including the pharynx and most of the gut successfully close the wound and survived up to the end of the observation period of six days, forming new unpigmented tissue at the wound site. This preliminary study was continued by Pechlaner [11], who chose five transversal cutting levels and included an additional experiment, in which the pharynx was amputated as a whole, while the rest of the animal was left untouched. Pechlaner saw great regeneration potential in *O. auditivum,* limited by a short life span (9 to 10 months) that would not allow sufficient time for complete regeneration at the low living temperatures (4 to 10°C). However, he was able to show the successful regeneration of the entire pharynx tube after its complete removal. Animals in which the pharynx was removed, by amputating the whole posterior part instead of the pharyngeal tube alone, were not able to regenerate the pharynx. Following anterior cuts, the anterior sensory pits with associated nerve cords could be regenerated, as well as major parts of the gut. Partial regeneration was observed for brain, eyes, yolk glands and gonads, but the natural end of the lifespan of the regenerates prevented full regeneration; the statocyst was never regenerated [11].

In the most recent work dealing with regeneration in proseriates, Giesa [8] cut *Monocelis fusca* just anterior to the pharynx. The anterior parts containing the brain regained full fertility after successfully regenerating their missing posterior body parts. Giesa mentions a similar regeneration capacity in the monocelidids *Coelogynopora biarmata*, *C. schulzii*, *Archilopsis unipunctata* and *Monocelis lineata,* and also in the otoplanids *Itaspiella armata* and *Bothriomolus balticus*[8].

In this study, we used a *Monocelis* species very similar to the *Monocelis fusca* depicted in [8]. *Monocelis fusca* is probably a species complex consisting of several distinct species [3], and is currently undergoing taxonomic revision (Marco Curini-Galletti, personal communication). For this reason, our experimental animals are referred to as *Monocelis* sp. With this species, we extended previous regeneration experiments with immunohistochemical and fluorescent imaging techniques. We amputated adult specimens anterior to the pharynx and visualized the response of the neoblast stem cell system, the serotonergic nervous system and the muscle filaments. In this way, a detailed picture of the regeneration process was obtained, allowing us insights into the cellular dynamics of the proseriate regeneration blastema, and the reorganization of the regenerating pharynx and tail plate.

Our study aims to explore the pattern of regeneration in a proseriate species, to allow for a better assessment of the evolutionary history of regeneration in the Platyhelminthes.

## Methods

### Specimen collection, animal culture and species identification

Experimental animals were collected at a coarse sand marine beach near the marine biological station Kristineberg in Fiskebäckskil, Sweden in July 2011 and 2013, and were extracted from the sand in the laboratory with 1:1 7% MgCl_2_.6H_2_O and artificial sea water (ASW) washed through a 40 μm mesh. Animals were maintained in the laboratory in Petri dishes in 3.2% ASW at room temperature (ca. 20 to 22°C) without feeding. Taxonomic identification was done on live squeeze preparations using a compound microscope.

### Artificial amputation and BrdU labelling

**Figure 1:**
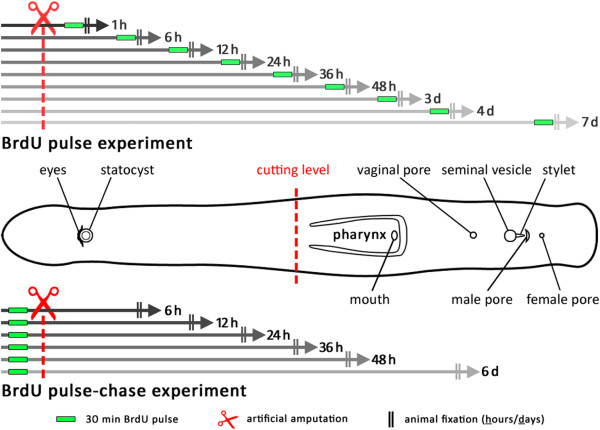
**Setup of BrdU pulse and pulse-chase experiments and schematic drawing of** ***Monocelis*** **sp.** In the pulse experiments, cells in S-phase were labelled *in situ* at nine different time intervals after amputation. Animals were fixed directly after labelling. In pulse-chase experiments, S-phase cells were labelled before amputation, were allowed to migrate and differentiate for five different time periods and were then fixed. The schematic shows an adult specimen (anterior to the left).

Animals were relaxed in 7% MgCl_2_.6H_2_O for about 15 min shortly before amputation, which was accomplished in a small droplet of ASW on a slide with a razor blade under a stereo microscope. A cutting level was chosen just anterior to the pharynx resulting in a posterior part (=anterior regenerate) that included the complete pharynx and an anterior part (=posterior regenerate) with the head, but without pharynx (Figure 1). Amputated specimens were transferred into new Petri dishes containing ASW and maintained at 20°C without feeding during the regeneration process. Anterior and posterior pieces were observed for at least 14 days.

S-phase cells were labelled by soaking specimens for 30 minutes in a 5 mM BrdU solution (Sigma-Aldrich, St. Louis, MO, USA) in ASW. Pulse-chase experiments allow us to follow the migration or differentiation of the BrdU-labelled cells in live animals (chase), whereas BrdU pulse experiments label S-phase cells *in situ* (no chase) (see [12]).Specimens designated for pulse-chase experiments were BrdU-labelled immediately before amputation and were subsequently allowed to regenerate for between 6 hours and 7 days before fixation. In our pulse experiments, specimens were amputated and allowed to regenerate for between 1 hour and 7 days and then soaked in a BrdU solution for 30 min and subsequently fixed (no chase) (Figure 1). In non-amputated BrdU pulse controls, animals were fixed immediately after BrdU soaking.

### Fixation

After reaching specific time points during regeneration (Figure 1, vertical double lines), animals were anaesthetized in 7% MgCl_2_.6H_2_O for about 15 min, fixed in 4% formaldehyde in 0.1 M PBS (60 min) at room temperature and rinsed with PBS (6 × 5 min). This was followed by a stepwise transfer through 25%, 50% and 75% methanol into 100% methanol. Animals were then stored at -20°C.

### Immunocytochemistry and fluorescence staining

BrdU-labelled specimens stored in methanol were rehydrated by several PBS-T (PBS with 0.1% Triton-X) washing steps for 60 min, then treated with 0.1 mg/ml proteinase K or proteinase XIV under visual control for about 60 min at room temperature, exposed to 2 M HCl for 60 min at 37°C (for animals of pulse experiments, pre-cooled 0.1 M HCl was used and animals were put on ice for 10 min), rinsed several times with PBS-T (for about 60 min) and blocked in BSA-T (PBS-T with 1% bovine serum albumin) for 30 min.

A monoclonal mouse anti-BrdU antibody (Developmental Studies Hybridoma Bank, G3G4) was used at 1:1000 (pulse-chase) or 1:500 (pulse) dilution in BSA-T overnight at 4°C. In pulse experiments, a rabbit anti-phospho-histone H3 antibody (Merck Millipore, Billerica, MA, USA) at 1:500 was added to the antibody solution. After washing in PBS-T (6 × 10 min) a secondary goat anti-mouse antibody (Alexa Fluor 488, Invitrogen) at 1:200 (pulse-chase) or 1:500 (pulse) in BSA-T was applied in darkness for 60 min at room temperature. In pulse experiments, a tetramethylrhodamine isothiocyanate (TRITC) conjugated swine anti-rabbit (DakoCytomation) secondary antibody at 1:300 was added to the antibody solution. After rinsing with PBS or PBS-T, animals were mounted on object slides in 80% glycerol or VectaShield (Vector Laboratories), sealed with nail polish and stored at -20°C. Controls for the BrdU and phospho-histone H3 staining were done by omitting primary or secondary antibodies.

To detect muscle actin filaments and serotonergic nerve cells, regenerating and control animals were relaxed, fixed in 4% formaldehyde (60 min), washed with PBS-T (6 × 10 min) and subsequently blocked with BSA-T (60 min) and incubated in a primary antibody solution (rabbit anti-serotonin 1:750, Sigma) in BSA-T at 4°C overnight. Animals were then washed with PBS-T (6 × 10 min) and blocked again with BSA-T (30 min). Animals were then incubated in a secondary antibody (TRITC-conjugated swine anti-rabbit 1:500) and phalloidin (Alexa Fluor 488 phalloidin, Invitrogen, 1:500) solution in BSA-T for 60 min at room temperature. Animals were finally washed with PBS-T (6 × 10 min) and incubated in PBS-T (60 min) before being mounted in VectaShield, sealed with nail polish and stored at -20°C. Controls for serotonin staining were done by omitting primary or secondary antibodies.

### Microscopy

The fluorescently stained and live animals were observed using a Zeiss Imager M1 and a Leica DM 5000 microscope. Confocal stacks were made on Leica TCS SPE and SP5 confocal microscopes.

### Sequences and phylogenetic analysis

We extracted total RNA from adult specimens, and the corresponding cDNA was sequenced on an Illumina HiSeq platform. We assembled the raw 100 bp paired end reads using Trinity v20131110 [13]. 18S and 28S ribosomal RNA sequences were identified with RNAmmer v1.2 [14] and have been submitted to GenBank (accession numbers KM457625 and KM457626).

For phylogenetic analysis, we downloaded available 18S and 28S ribosomal RNA sequences of proseriates from GenBank (Additional file 1) and separately aligned 18S and 28S sequences using Clustal Omega [15]. The 18S and 28S alignments were concatenated, ambiguous alignments were pruned with trimAl using default parameters [16] and a maximum likelihood phylogenetic tree was reconstructed with PAUP* v4.0b10 [17]. An initial tree was estimated using neighbor joining. This initial tree was used to estimate the parameters of the likelihood model (generalized time reversible substitution matrix and gamma parameter) and a heuristic tree bisection and reconnection search, starting from the original neighbor joining tree, was performed using the previously calculated parameters. The tree was rooted with the branch encompassing all included Unguiphora (*Nematoplana*, *Polystyliphora*).

### Ethical approval

Flatworms are not regulated in directive 2010/63/EU of the European Parliament or the UK Animals (Scientific Procedures) Act 1986, but care has been taken to minimize potential suffering of animals.

## Results

For all stages (controls and anterior and posterior regenerates), we have labelled more than 220 animals, about 200 of which were artificially amputated. In total, we made 279 confocal stacks of 115 different specimens. Additionally, about 40 animals were used for live observations.

### Phylogenetic analysis

Our phylogenetic tree based on 18S and 28S ribosomal RNA genes suggests a close relationship between the *Monocelis* species from the present study and *Monocelis lineata*, followed by *Monocelis fusca* and *Monocelis longistyla* (Additional file 2).

### Intact animals

**Figure 2:**
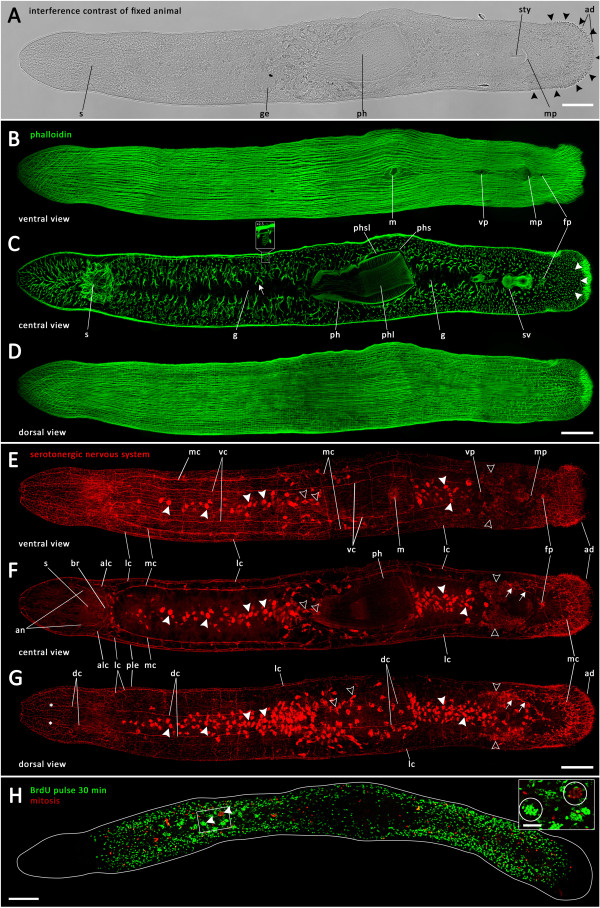
**Intact animals of** ***Monocelis*** **sp. (A)** Differential interference contrast image. The distribution of the adhesive system is indicated by black arrowheads. **(B)** Ventral, **(C)** central and **(D)** dorsal views of phalloidin-stained actin filaments. White arrow in **(C)** points at a forked muscle fibre, white arrowheads demarcate the position of the caudal nerve loop. Inset shows a magnified paracnid-like cell. **(E)** Ventral, **(F)** central and **(G)** dorsal view of the serotonergic nervous system. White arrows in **(F-G)** indicate female pore-related gland cells; white arrowheads indicate gut-related gland cells; hollow arrowheads indicate seminal vesicle-related gland cells. Asterisks in **G** mark the anterior end of dorsal nerve cords. **(H)** 30 min BrdU-pulse-labelled S-phase cells (green) and mitoses (red); inset with presumptive testis encircled. ad, adhesive system; alc, anterior lateral nerve cords; an, anterior nerves; br, brain; dc, dorsal nerve cords; fp, female pore; g, gut; ge, germaria; lc, lateral nerve cords; m, mouth; mc, main nerve cords; mp, male pore; ph, pharynx; phl, pharynx lumen; phs, pharynx sheath; phsl, pharynx sheath lumen; ple, plexus; s, statocyst; sty, stylet; sv, seminal vesicle; vc, ventral nerve cords; vp, vaginal pore. Anterior is left in all panels. Scale bar, 100 μm; inset, 25 μm.

**Figure 3:**
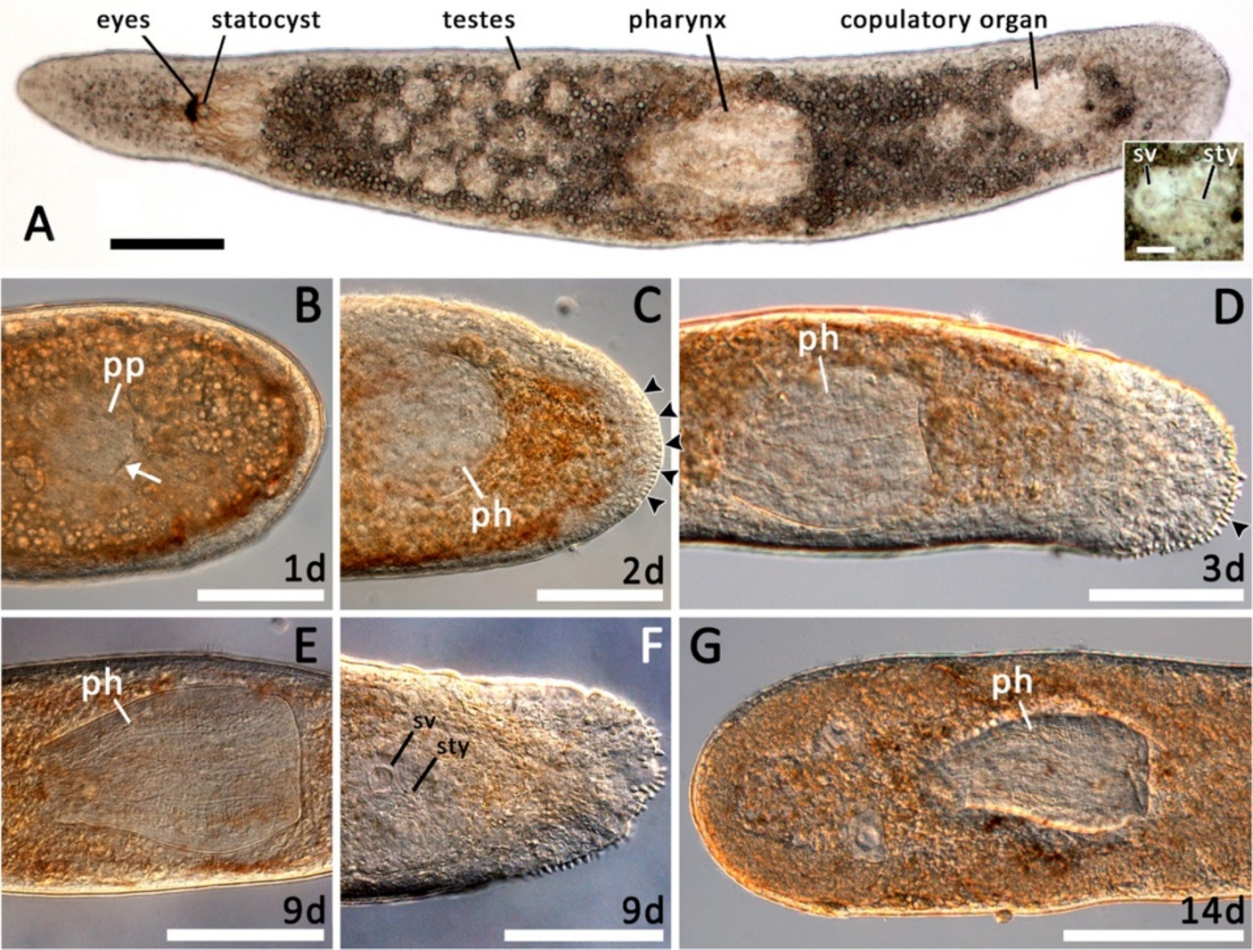
**Live observations of** ***Monocelis*** **sp. (A)** Overview of a live animal with fused pigmented eyespots, statocyst, testes, pharynx and copulatory organs. The inset shows the seminal vesicle and the stylet. **(B-F)** Posterior regenerates with regenerating pharynx and tail at different time points. **(B)** One-day-old regenerate showing an unpigmented area, the pharynx primordium (pp). The white arrow indicates the formation of the pharyngeal sheath. The wound is already closed. **(C)** Two-day-old posterior regenerate with a spherical immature pharynx and first signs of regeneration of the duo-gland adhesive systems (black arrowheads). **(D)** Three-day-old posterior regenerate with elongated, fully functional pharynx and additional duo-gland adhesive systems (black arrowhead). **(E, F)** 9-day-old posterior regenerate showing a fully regrown pharynx and seminal vesicle and stylet. **(G)** 14-day-old anterior regenerate with its original pharynx and no sign of any ongoing regeneration process. ph, pharynx; pp, pharynx primordium; sty, stylet; sv, seminal vesicle. Scale bars, 100 μm; inset, 25 μm. All images with anterior to the left.

To provide a baseline for the regeneration studies, we first investigated the general anatomy of intact *Monocelis* sp. with a focus on the musculature, the nervous system and proliferating cells. A schematic overview of *Monocelis* sp. is given in Figure 1. The rostrum is the anterior-most part of the animal, including merged eyes, statocyst and brain. The gut extends from behind the brain to the tail, the rearwards-pointing pharynx is situated slightly posterior of the middle. Copulatory organs are situated posterior to the pharynx. Adult specimens were found to have a stylet (male copulatory organ) with a posteriorly orientated tip with an average length from base to tip of about 50 μm (48.9 μm ± 3.9 μm, *n* = 12) (Figures 2A, and 3A inset). The duo-gland adhesive systems are situated at the tip of the tail (Figure 2A, black arrowheads).

Body wall musculature revealed by phalloidin-stained F-actin consists of a prominent ventral and dorsal longitudinal layer (Figure 2B, D), which is surrounded by a much thinner outer circular layer. Diagonal muscles appear to form the most external layer (Additional files 3 and 4). Additionally, the animal’s parenchyma is crossed by numerous dorsoventral muscles, also revealing the loop of the main nerve cord within the tail plate, seen as an empty space (Figure 2C, white arrowheads). The brain of *Monocelis* sp. is traversed by and encapsulated within many thick muscle fibres (Figure 2C). About 100 blunt-ended, broadly spindle-shaped structures (22.9 μm ± 3.4 μm in length and 9.1 μm ± 0.9 μm in width at their broadest point; *n* = 8) with a basket-like, phalloidin-positive surface could be seen in the central view of the animals, distributed along the anteroposterior axis from in front of the statocyst back to the tail plate, predominantly at the sides of the animals (Figure 2C, inset). Ventral longitudinal muscles are interrupted by several centrally aligned body openings (Figure 2B). The anterior-most body opening is the mouth, followed by the vaginal pore and then, close together, the male pore and the female pore (Figure 2B). The central view (Figure 2C) allowed insight into different organs of intact specimens, including the brain enclosed within and traversed by thick muscle fibres, the lumen of the gut, which is surrounded by numerous thicker forked muscle fibres (Figure 2C, white arrow) and the seminal vesicle. Starting approximately in the middle of the animal’s body and embedded in a delicate sheath lies the pharynx with prominent musculature beneath the outer and inner pharynx epithelia, the latter enclosing the pharyngeal lumen (Figure 2C). No opening could be discerned on the dorsal surface (Figure 2D).The serotonergic nervous system is organized in an orthogon consisting of pairs of main, lateral, dorsal and ventral longitudinal nerve cords (Figure 2E-G). The main nerve cords are located between the lateral and the ventral cords and could be seen in ventral to mid-ventral confocal images (Figure 2E, F). The nerve cords run posteriorly from the brain region and form a loop in the caudal region of the animal (Figure 2F). The lateral nerve cords reach from the anterior tip of the animal, where they almost merge, into the brain and extend from the brain to the tail plate, where they join the caudal loop of the main nerve cords (Figure 2F). The ventral and dorsal nerve cords extend in a similar way from the brain region, but run both ventrally and dorsally near the body surface (Figure 2E, G). Along the anteroposterior axis, the ventral cords are connected with the main nerve cords through about 20 transverse commissures going through the middle. The dorsal nerve cords are similarly connected with each other and with the lateral nerve cords by about 20 transverse commissures (Figure 2E-G). The ventral cords can be followed posteriorly to about the level of the vaginal pore (Figure 2E), while the dorsal cords extend posteriorly only to about the level of the mouth (Figure 2G).A fine subepidermal nerve plexus covers the whole animal, with the notable exception of the centre of the anterior- and posterior-most tips (Figure 2E, G). In centre-view confocal images, the plexus can appear like marginal 'nerve cords' (Figure 2F).At the tip of the tail, a dense network of serotonergic nerve fibres could be detected (Figure 2E-G).

Mitoses and BrdU-labelled S-phase cells of intact *Monocelis* sp. show a rather even distribution in two broad longitudinal stripes throughout the whole animal, with the exception of the rostrum (the region anterior of the brain), the epidermis, the pharynx and the tip of the tail (Figure 2H). The lack of neoblasts in the gut region is recognizable as a darker midline band. The head region from the anterior tip to the brain region is entirely free of S-phase cells and mitoses, as is the posterior tip of the tail plate.Conspicuous clusters of proliferating cells could be seen in testis follicles, which are located on both sides of the animal between head and pharynx. (Figure 2H, white arrowheads; encircled in inset).During image acquisition via confocal laser scanning microscopy, four different types of gland cell became apparent, which were also present in stained control animals, where only either primary or secondary (TRITC-conjugated) antibodies were applied. Positioned around the intestinal lumen with their highest abundance at the dorsal side near the gut (Figure 2E-G, white arrowheads), the staining of these gland cells should not be confused with serotonergic staining of nerve cells. A second type of gland cell was observed primarily in patches within the pharyngeal region, but appeared to accumulate around the male gonads (Figure 2E-G, hollow arrowheads). Furthermore, narrow gland cells, apparently associated with the female pore, were stained (Figure 2F-G, white arrows). Lastly, at the tip of the tail, the duo-gland adhesive systems were stained (Figure 2E-G).

### Regeneration

#### Live observations

After 24 hours, posterior regenerates had already formed the pharyngeal primordium, within which the pharyngeal sheath could be observed (Figure 3B, white arrowhead). After 48 hours, the primordium had already transformed into a spherical, immature pharynx (Figure 3C) and additionally, animals started to show first signs of the adhesive system at the posterior tip (Figure 3C, black arrowheads). After 3 days, the adhesive system of posterior regenerates was functional and the completely regenerated pharynx showed no major differences from that of intact animals (compare Figure 3D and E). After 8 days of regeneration, parts of the copulatory organ could be observed within the tail (data not shown), and after 9 days, structures such as the vaginal pore, the seminal vesicle and the stylet could be distinguished (Figure 3F). A putative male pore could be observed 10 days after regeneration (data not shown).Anterior regenerates showed no noticeable regeneration; even 14 days after artificial amputation, the head had not regenerated (Figure 3G).

#### Pulse experiments in anterior fragments (posterior regenerates)

**Figure 4:**
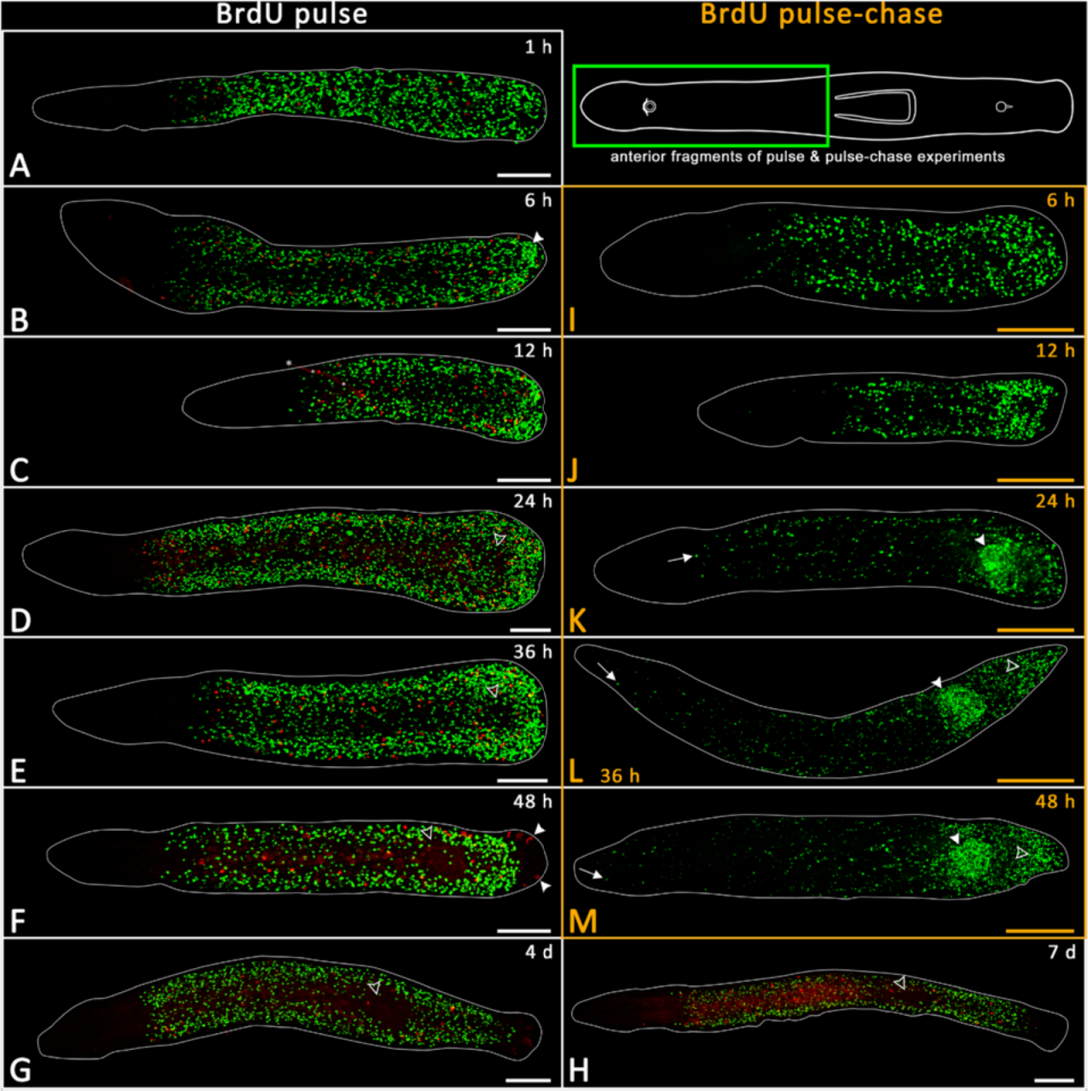
**BrdU pulse and pulse-chase experiments with posterior regenerates at different time points.** BrdU-labelled cells in green; mitoses in red; anterior is to the left. **(A-H)** BrdU pulse experiments are framed in white. **(I-M)** Pulse-chase experiments are framed in orange. **(A-E)** Pulse experiments reveal proliferation of BrdU-labelled cells at the posterior end (blastema) at 6 to 36 hours of posterior regenerates and show the reliance of the regenerating pharynx on proliferating cells around but never within its primordium **(D-F)**. This absence of proliferation is highlighted by hollow arrowheads. **(K-M)** BrdU pulse-chase experiments showing the specific migration of BrdU-labelled cells into the pharynx primordium between 24 and 48 hours, indicated by white arrowheads. Asterisk in **C** denotes an artefactual red line. Scale bars, 100 μm.

At 1 hour after amputation, the distribution of BrdU-labelled S-phase cells and mitoses was similar to control animals, with no noticeable accumulation at the wound site (Figure 4A). At just 6 hours post-amputation, an aggregation of proliferating cells arose in the very posterior part of the animals (Figure 4B, white arrowhead). In 12-hour and 24-hour regenerates, the accumulation of proliferating cells at the caudal end continued (Figure 4C-D). Additionally, 24-hour regenerates showed a distinct absence of labelled S-phase cells and mitoses within the region of the pharynx primordium (Figure 4D, hollow arrowhead). In animals that had been allowed to regenerate for 36 hours, S-phase cells and mitoses were not observed within the pharyngeal primordium, but were abundant around the pharyngeal primordium and continued to aggregate at the caudal end of the body (Figure 4E, hollow arrowhead). A few S-phase labelled cells seemed to appear within the pharynx primordium, but careful observation showed that they actually lay in the mesenchymal space either below or above the primordium. At 48 hours post-amputation, the newly developed pharynx was clearly recognizable as a slightly elongated proliferation-free region (Figure 4F, hollow arrowhead). The posterior tip of the regenerate was also free from proliferation. In animals that had been allowed to regenerate for 3 days (data not shown) and 4 days, there was no apparent additional accumulation of proliferating cells (Figure 4G). Similarly, in 7-day-old regenerates, no accumulated proliferating cells in the posterior region could be observed (Figure 4H).

#### Pulse-chase experiments in anterior fragments (posterior regenerates)

To track the migration and differentiation of BrdU-labelled cells, a series of pulse-chase experiments was performed (Figure 1). After 6 hours of regeneration, animals started to show a slight aggregation of labelled cells in the posterior part of their body (Figure 4I). In 12-hour regenerates, pulse-chase-labelled cells could be seen to a greater extent within the posterior region of the regenerates (Figure 4J). After 24 hours, labelled cells had clearly accumulated within the pharyngeal primordium (Figure 4K). Additionally, anteriorly migrated labelled cells became apparent in the head region (Figure 4K, white arrow). 36 hours post-amputation, posterior regenerates had such a high number of pulse-chase-labelled cells within the pharyngeal primordium that it was even possible to discern the shape of a newly developing pharynx (Figure 4L, white arrowhead). At the same time, pulse-chase-labelled cells became concentrated towards the caudal end of the animals (Figure 4L, hollow arrowhead). Compared with 24-hour regenerates, the anterior movement of BrdU-labelled cells into the head region became even more noticeable at 36 hours and 48 hours (Figure 4L-M, white arrow).

#### Determining migration speed of BrdU-labelled cells

In BrdU pulse experiments, the distance from the anterior-most BrdU-labelled cell to the tip of the rostrum was 240.7 μm ± 27.8 μm (*n* = 9). In BrdU pulse-chase experiments, BrdU-labelled cells (almost) reached the tip of the rostrum after 48 hours of regeneration (Figure 4M), suggesting a migration speed of BrdU-labelled cells of about 5 μm per hour.

#### Pulse and pulse-chase experiments in posterior fragments

**Figure 5:**
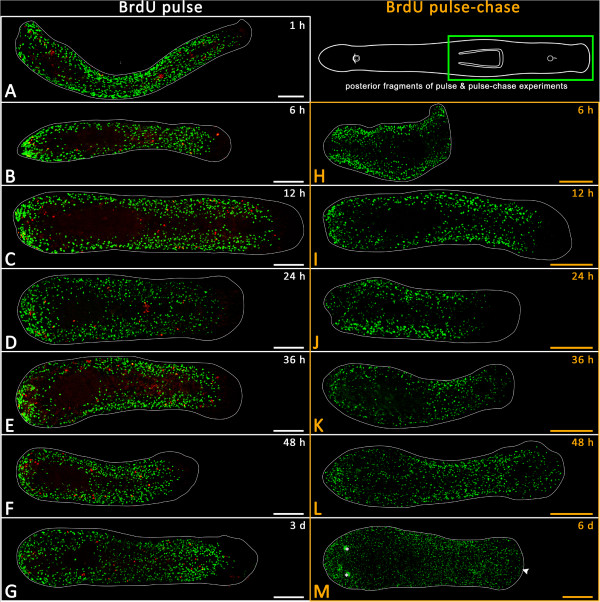
**BrdU pulse and pulse-chase experiments with anterior regenerates at different time points.** BrdU-labelled cells in green, mitoses in red; anterior is to the left. **(A-G)** BrdU pulse experiments are framed in white. **(H-M)** Pulse-chase experiments are framed in orange. **(B-F)** Anterior ends of posterior fragments show increased cell proliferation activity close to the wound site between 6 hours and 48 hours post-amputation. However, no head was regenerated. **(I-M)** The pulse-chase experiments tracked the gradual migration of BrdU-labelled cells into the tail plate. The two green spots in **(M)**, indicated by white asterisks, are artefacts. Scale bars 100 μm.

In pulse and pulse-chase experiments, the anterior ends of posterior fragments were unable to regenerate any anterior organs but, as with posterior pieces of pulse-chase experiments, a weak aggregation of BrdU-labelled cells could often be observed at the anterior end (Figure 5B-G,I-M).In pulse-chase experiments, after 24 hours of regeneration a posterior migration process of BrdU-labelled cells became apparent (Figure 5J) and even more so in 36-hour-old regenerates, in which the migration process had advanced almost to the posterior end of the tail plate (Figure 5K). The posterior part of the tail plate became completely populated by BrdU-labelled cells after 48 hours and at later time points (Figure 5L-M, white arrowhead).

#### Phalloidin staining in anterior fragments

**Figure 6:**
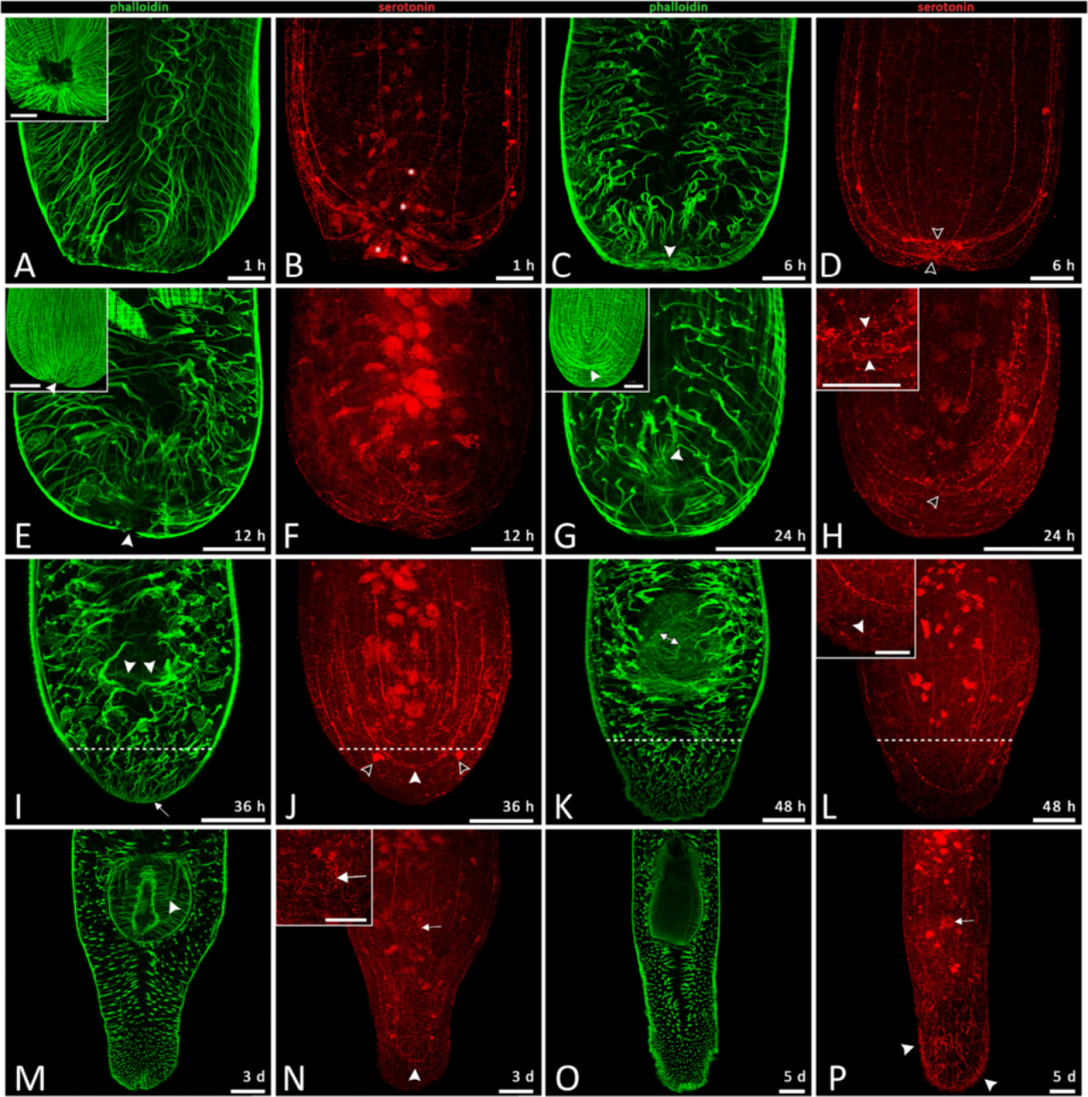
**F-actin musculature (green, first and third column) and serotonergic nervous system (red, second and fourth column) in posterior regenerates; anterior is up. (A, B)** 1 hour post-amputation: constricted ring musculature, wound not yet closed. A few autofluorescent gland cells are marked with white asterisks. **(C, D)** 6 hours: fine network of muscle fibres covers wound (white arrowhead); main nerve cords start reorganizing (hollow arrowheads). **(E, F)** 12 hours; advanced wound closure (white arrowhead), serotonergic fibres are comparable to 6-hour-old regenerates. **(G, H)** 24 hours: wound closure by muscle fibres is complete (inset in **G**), former wound is still visible as small serotonergic ring (arrowheads in **(H)** and inset). **(I, J)** 36 hours: first pharyngeal actin filaments (note fine muscle fibres beneath two white arrowheads); blastema area can be estimated by weaker staining of fine muscle network at posterior site (dashed line); new nerve cell bodies (hollow arrowheads) appear in blastema, and nerve cords are connected (white arrowhead in **J**). **(K, L)** A spherical immature pharynx connected to the epidermis by strong muscle fibres appears, and lumen forms (white double arrow); blastema increases (dashed line). Fine serotonergic nerve fibres can be spotted in the most posterior part (white arrowhead, inset in **L**). **(M, N)** Tail and pharynx formation has significantly advanced in the last 24 hours. **(M)** White arrowhead indicates fissures of transverse muscles beneath outer pharynx epithelium. **(N)** Serotonergic staining shows nerve fibres within tail plate (white arrowhead) and of mouth (white arrow, detail in inset). **(O, P)** Regeneration of pharynx and tail are complete. Adhesive systems cover most of tail (see white arrowheads). Mouth nerve fibres are clearly visible (white arrow). Copulatory structures are still missing. Scale bar in **(A-P)** 50 μm; scale bar in **(A)** and **(E)** insets 50 μm; scale bar in **(G)**, **(H)**, **(L)** and **(N)** insets 25 μm.

At 1 hour after amputation, phalloidin staining showed a rather small wound opening, owing to the constriction of circular muscles (Figure 6A inset). At 6 hours, a finer network of muscle fibres had appeared and covered the wound (Figure 6C, white arrowhead). At 12 hours post-amputation, the wound closure had become more complete and the posterior ends of the animals acquired a more rounded shape than at previous time points (Figure 6E). The wound was still not entirely sealed by musculature, however, and was therefore clearly recognizable (Figure 6E, white arrowhead). After 24 hours of regeneration, the covering of the wound by fine muscle fibres was added to by more substantial muscle fibres, and the wound site was now completely covered (Figure 6G, white arrowhead). Fine muscle fibres became concentrated within the region of the pharyngeal primordium (Figure 6G, white arrowhead). At 36 hours after amputation, longitudinal muscle fibres from the trunk had projected thin extensions posteriorly (Figure 6I, white arrow), and longer, horizontal pharyngeal F-actin filaments appeared in the region of the pharyngeal primordium (Figure 6I, white arrowheads). After 48 hours of regeneration, a globular early pharynx was apparent (Figure 6K). Simultaneously, the formation of the inner pharynx epithelium, which is delineated by actin fibres, took place (Figure 6K, white double arrow) and the mouth opening became visible (data not shown). One day later, it became clear that the orientation of the pharynx up to this point had still been vertical, and only now started to tilt towards a horizontal orientation (Figure 6M, Additional file 5). Transverse fibres reinforced the outer pharynx epithelium (white arrowhead points at a fissure in the fibres), and the inner pharynx epithelium enclosing the pharynx lumen took shape within a mesh of longitudinal and transverse fibres (Figure 6M). A complete pharynx was obvious after five days (Figure 6O), virtually identical to that of intact control animals of *Monocelis* sp. (compare Figure 2C).

**Figure 7:**
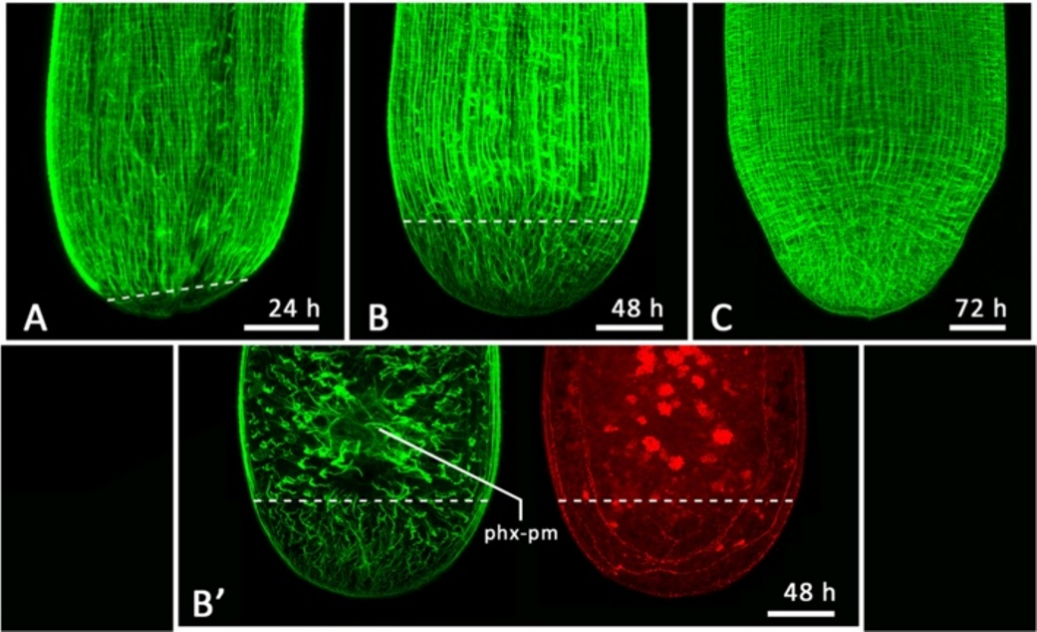
**Blastema dimensions. (A)** F-actin staining of a posterior regenerate 24 hours post-amputation reveals first signs of a small blastema at the tail tip of the specimen (beneath the dashed line) **(B)** F-actin staining of a 48-hour post-amputation posterior regenerate shows a more advanced blastema delineated by a dashed line. **(C)** F-actin staining of a posterior regenerate 72 hours post-amputation. At this point of regeneration, a blastema is no longer recognizable. **(B′)** Same animal as **(B)**. Central view of actin fibres reveals that the pharynx primordium is lying outside the blastema. Serotonergic staining shows the already successfully reconnected main nerve cords. Dashed lines indicate the estimated blastema size. phx-pm, pharynx primordium.

Also visible in 36- and 48-hour regenerates was the extent of regenerated and regenerating tissue at the posterior end, which could be estimated by phalloidin label intensity and disorderly actin fibre arrangement (dashed line in Figure 6I, K). While the pharynx was formed in the uninjured tissue anterior to the wound site, the animal had elongated by 33.0 μm ± 10.0 μm (*n* = 5) with newly formed tissue after 36 hours and by 78.1 μm ± 15.3 μm (*n* = 6) after 48 hours. We define this new tissue as the blastema sizes at the respective time points. After 3 days of regeneration, the actin fibres in the posterior end were indistinguishable from those in the rest of the body (Figure 7).

A peculiar observation was made in animals at later time points (5 to 14 days). Six out of 19 posterior regenerates were unable to regenerate the pharynx properly, or were seriously delayed in doing so. The aberrant pharynges appeared as oversized clusters of muscle fibres, often with little or no organization (Additional file 6).

#### Phalloidin staining in posterior fragments

In anterior regenerates F-actin staining did not reveal any regeneration process except wound closure due to constriction of circular muscles and subsequently the merging of longitudinal muscle fibres at the wound site (Additional file 7).

#### Staining of the serotonergic nervous system in anterior fragments

After characterizing the nervous system of intact animals, we investigated its reorganization during regeneration. At 1 hour post-amputation, the severed serotonergic nerve cords became constricted by circular muscles located around the wound (Figure 6B). After 6 hours of regeneration, the serotonin staining showed a rather unstructured net of nerve fibres at the wound site, but the main nerve cords had already started a process of reorganization and reconnection (Figure 6D, hollow arrowheads).Serotonergic nerve cells in 12-hour and 24-hour regenerates looked similar to 6-hour regenerates, but the main nerve cords were less tangled (Figure 6F,H). In 24-hour posterior regenerates, a small ring, formed by the end of the previously severed nerve cords, still indicated the place of the former wound caused by artificial amputation (Figure 6H, hollow arrowhead). At 36 hours after amputation, the disentanglement and reconnection of the main nerve cords was complete (Figure 6J, white arrowhead). Posteriorly situated nerve cell bodies started to emerge within the very posterior part of the animals (Figure 6J, hollow arrowheads). At 48 hours post-amputation, animals already showed a few serotonergic nerve fibres within the blastema (Figure 6L inset, white arrowhead).Within the tail plate of a 3-day-old posterior regenerate, several more serotonergic nerve fibres could be observed (Figure 6N, white arrowhead). The presence of a mouth was indicated by a corresponding circle of nerve cells (Figure 6N and inset, white arrow). After animals had regenerated for 5 days, numerous adhesive organs had appeared and these covered a much greater part of the tail plate than in the 3-day old regenerates (Figure 6P, white arrowheads). Nerve cells of the pharynx sheath opening were obvious (Figure 6P, white arrow) and a dense network of nerve fibres was visible within the regenerated tail plate (Figure 6P).

#### Staining of the serotonergic nervous system in posterior fragments

The staining of the serotonergic nervous system at the wound site was very weak, but in 24-hour regenerates at least, the main nerve cords seem to have merged (Additional file 7B). No further regeneration processes could be observed.

## Discussion

### Species determination

The animals used in this study conformed in large parts to the species description of *Monocelis fusca*[3, 18, 19]. However, the stylet of *M. fusca* points anteriorly, while, in our species, the stylet points posteriorly (Figure 2A). Interestingly, Giesa [8] calls the species used in his studies *M. fusca*, even though the drawing he provides reveals a posteriorly orientated stylet. This discrepancy supports the notion that *M. fusca* is in fact a species complex of very similarly looking species [3]. *Monocelis longistyla* is another similar looking species with a backwards pointing stylet more than 100 μm long, but is known only from the Mediterranean [19].

Our phylogenetic tree clearly supports the species used in the present study being a *Monocelis* species, but interestingly *Monocelis lineata*, which does not have a stylet, is shown to be the closest related species (Additional file 2). A phylogenetic tree using more representatives of the genus *Monocelis* from several geographical locations may shed more light on the interrelationships of *Monocelis* species.

### Regeneration capacity

**Figure 8:**
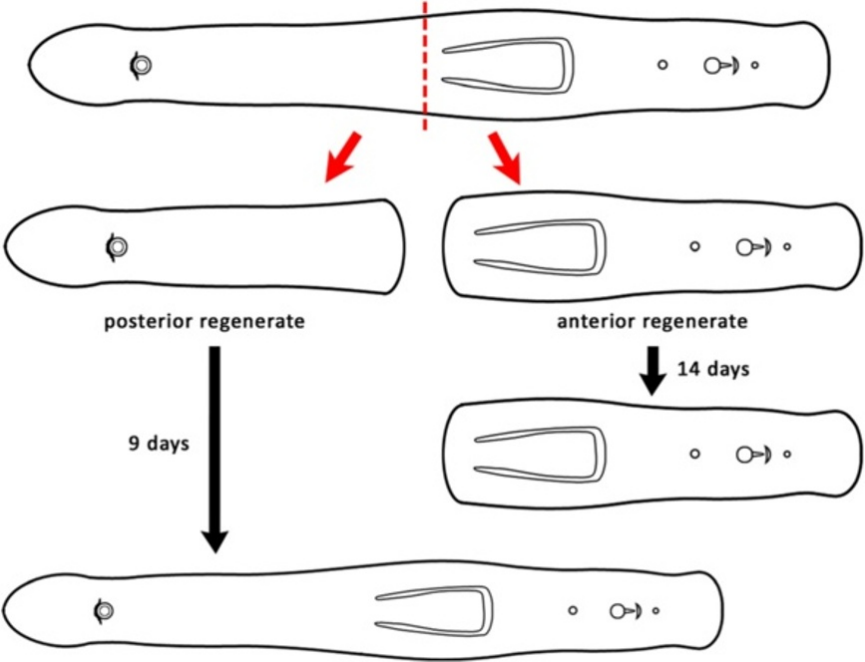
**The regeneration capacity of** ***Monocelis*** **sp.** The posterior regenerate can regenerate the missing parts within approximately 9 days. The anterior regenerate without head cannot regenerate a head or any other anterior organs.

The single previous publication dedicated to regeneration in proseriates deals with the freshwater proseriate *Otomesostoma auditivum*[11]. In a posterior fragment cut behind the pharynx, a pharynx was partially regenerated, showing that the presence of a brain is not required for pharynx regeneration in this species. Interestingly, in anterior regeneration experiments, if cut at or just behind the brain, *O. auditivum* can at least partially regenerate the eyes, brain and anterior sensory pits (after 99 days of regeneration), but not the statocyst [11]. The cutting level just anterior to the pharynx employed in [8] and our study did not show any signs of regeneration of brain, eyes or statocyst in the posterior fragments (Figure 8), but cutting levels just posterior of the brain would be interesting to test for the possibility of regenerating the brain in *Monocelis*.

A relationship between reproduction mode and regeneration capacity in flatworms has been noted, in that obligatory sexually reproducing species often have a reduced regeneration capacity compared with asexually reproducing species [7, 20]. Proseriates conform to these expectations in that all proseriates have obligatory sexual reproduction, and they cannot regenerate the head [7]. In triclads, where many sexually reproducing species can regenerate their head, this relationship is less pronounced than in other flatworms [21].

We now have data from several representatives of four families of the Proseriata Lithophora (proseriates bearing a statocyst), whereas reports concerning regeneration capacity from members of the statocyst-less Proseriata Unguiphora (ca. 40 species [22]) are still lacking.

### Speed of regeneration

Pechlaner [11] performed a number of regeneration experiments with the freshwater proseriate *Otomesostoma auditivum* (Otomesostomidae) and concluded that the species’ slow and limited regeneration process is due to its cold stenothermal environment of 4 to 10°C and its limited life span of 9 to 10 months. As an example, it takes anterior fragments of *O. auditivum* 71 days to regenerate the parts posterior of the pharynx, and even after this time the gonads are only partially restored. Anterior fragments with an amputated pharynx are able to regenerate a functional pharynx within 9 to 10 weeks [11]. In *Monocelis fusca, Monocelis lineata*[8] and our *Monocelis* sp., anterior pieces also readily regenerate the pharynx within a few days at about 20°C. The same is true of *Pseudomonocelis* sp. (own observations, data not shown) and *Archilopsis unipunctata*[8] (all belonging to the family Monocelididae), two species of *Coelogynopora* (Coelogynoporidae) and *Itaspiella armata* and *Bothriomolus balticus* (both Otoplanidae [8]).

Besides the pharynx, in our study *Monocelis* sp. also regenerated the duo-gland adhesive system after 2 or 3 days and the genital organs after 8 to 10 days, as well as the stem cell system, the musculature and the nervous system. This corroborates findings that posterior regeneration in *Monocelis* is indeed a complete regeneration of all missing body parts. In tail plate regenerates of *Macrostomum lignano*, the first duo-gland adhesive organs appear two days after amputation [23]. The pronounced difference in regeneration speed between *Otomesostoma* and the other proseriates tested may be attributed either to the difference in culture temperatures or to the different species used, or to both. This question could be addressed by letting *Monocelis* sp. regenerate at lower temperatures, as this species naturally occurs in the cold waters of the Skagerrak with a measured mean annual sea surface temperature between 9 and 11°C [24].

### Migration speed of BrdU-labelled cells

In triclads, cell migration speed has been determined to be about 40 μm per day [25], which is about 1.7 μm per hour. In *M. lignano*, the cell migration of BrdU-labelled cells is about 6.5 μm per hour [26], similar to the 5 μm per hour we found for *Monocelis* sp. In catenulids, no migration speed was determined, but judging from the figures given in [27, 28] using the same method described in this paper, the migration speed is about 4.7 μm per hour (*n* = 3).

BrdU-labelled cells migrating anteriorly, away from the blastema, in posterior regenerates are probably contributing to cell homeostasis, and not to regeneration.

### Cell proliferation and blastema formation

We have observed proliferating cells (in S-phase or mitosis) in two broad stripes along the anteroposterior axis in both intact control (Figure 2H) and regenerating animals (Figures 4 and 5). The absence of proliferation anterior to the brain and in the epidermis has been shown for other flatworms as well, for example *Paracatenula*[27, 28], *Macrostomum*[23, 29] and several triclads [30], the biological significance of which is still unclear. During posterior regeneration, proliferating cells begin to accumulate at the wound site about 6 hours after amputation (Figure 4B). Proliferation continues in the posterior-most part of the animals until 36 hours after amputation (Figure 4C-E). From 48 hours onwards, the proliferation in the posterior-most tip of the tail plate ceases and resembles control animals, in which no proliferation is detectable in the posterior tip of the tail plate (Figure 2H). We define the time span from 6 to 48 hours after amputation as being the period of blastema formation. A blastema is characterized by the accumulation of undifferentiated cells at the wound site, covered by epithelium [12, 23].

### Proliferation within the blastema proper

Unlike in triclads [31, 32], but similar to catenulids [27, 28] and macrostomorphans [23], we could not observe a postblastema in regenerating proseriates. The postblastema is adjacent to (and in anterior regeneration posterior to) the blastema and is a centre of proliferation in triclads, in which no proliferation in the blastema proper occurs [32]. In all other flatworms where cellular dynamics during regeneration have been studied, the blastema is also a centre of proliferation ( [23, 27, 28] and this study). Given that the systematic position of the Catenulida, Macrostomorpha and Proseriata appears to be more basally branching within the Platyhelminthes than the Tricladida [33, 34] it is conceivable that the condition found in triclads (no proliferation within the blastema) is derived and that the plesiomorphic condition for platyhelminths is the presence of proliferation within the blastema. Investigations in other flatworm groups will provide a clearer picture.

### Pharynx regeneration relies on cell migration and proliferating cells

Pharynx formation in *Monocelis* sp. took place anterior to and outside of the blastema (Figure 7). Our pulse-chase experiments showed that BrdU-labelled cells specifically migrated into a pharyngeal primordium and in this manner supplied it with fresh cell material (Figure 4K-M). Conversely, there was a notable absence of proliferating cells *in situ*, that is, within the pharynx primordium (Figure 4D-F). This situation is strikingly similar to *M. lignano*, where it was shown that neoblasts do not proliferate at the site of differentiation of the regenerating genital apparatus [23].

### F-actin

As a means to compare the regeneration of the musculature in *Monocelis* with intact animals, we have documented for the first time the complete F-actin complement in a proseriate species. Although phalloidin staining was already used for the species description of an otoplanid proseriate [35], these did not reveal the overall pattern of F-actin fibres. Our findings showed the arrangement of F-actin fibres in unprecedented detail and provide several new insights: (1) the diagonal fibres may be the outermost muscle layer, or at least they seem to interleave with ring muscle fibres (Additional files 3 and 4). This is very unusual and deviates from the common pattern of outer circular, medium diagonal and inner longitudinal musculature in platyhelminths [36, 37]. (2) Spindle-shaped actin fibres (Figure 2 inset, Additional files 8 and 9) are present in *Monocelis* sp. and are reminiscent of the so-called paracnids found in the proseriate species *Coelogynopora axi*, which are micro-organs consisting of a muscle cell surrounding a gland cell [38]. In image stacks of phalloidin-labelled *Monocelis* sp., we could follow the phalloidin-labelled spindles to a small, but distinct opening within the epidermis (Additional file 9), hinting at a secretory cell type such as a gland cell within. This is the first report of muscle-wrapped gland cells in a proseriate outside of the Coelogynoporidae.

In another microturbellarian, *Macrostomum* sp., the posterior regeneration of the musculature happens in a very similar manner and time frame as in *Monocelis*, although in the latter species the wound did not shift to the ventral side during early regeneration as it does in *Macrostomum*[23, 39]. In both species, the intact muscle fibres send out fine projections into the wound site and these gradually extend and become broader. This behaviour also matches observations in the triclad *S. mediterranea*, although here anterior regeneration was studied and the regeneration process takes more time than in the smaller flatworms [40].

### Similarities between embryonic development and regeneration

The complete embryonic development in *Monocelis fusca* from egg laying to hatching takes 5 to 7 days, depending on temperature [8]. Pharynx formation during embryonic development starts with the vertically orientated pharynx anlage (primordium), which subsequently tilts by 90°, so that the pharynx lies horizontally along the main body axis. The inner pharyngeal epithelium forms first, and this then separates along the central longitudinal axis, thereby forming the pharyngeal lumen. Later, the outer pharyngeal epithelium continues from the edge of the inner pharynx epithelium. The pharyngeal sheath initially develops without connection to the body epithelium and later joins the invaginated epidermis to form the pharynx sheath opening (mouth).

Our observations of regeneration in *Monocelis* sp. match the order of events during pharynx organogenesis in developing embryos of *M. fusca.* An early step in *Monocelis* sp. regeneration is the formation of the inner pharynx epithelium (Figure 6K) that emerges within an already recognizable bulbous, vertically orientated pharyngeal primordium about 48 hours post-amputation (see also Figure 3C). The pharynx primordium starts to tilt towards the horizontal axis about 3 days post-amputation (Figure 6M) and this is followed by its elongation (Figures 3D-E, 6O). The fissures seen in the transition stage (Figure 6M) may be necessary to allow the vertical pharynx to flip over horizontally. Also similar to embryonic development, pharynx regeneration took about 5 days until completion.

### Is a vertical pharynx primitive for proseriates?

The Proseriata are subdivided into the Unguiphora (without statocyst) and the Lithophora (with statocyst) [2]. The Unguiphora are predominantly equipped with a vertically directed pharynx, unlike most Lithophora, which have a horizontal pharynx [22]. Although the orientation of the pharynx seems to be highly variable in proseriates, with both orientations present in different species of some genera (for example, *Nematoplana* and *Invenusta*) [2, 22], we propose that the plesiomorphic condition in proseriates is a vertically orientated pharynx, reflected by the pharynx organogenesis, in which a vertical pharynx arises first, even in a species which eventually has a horizontal pharynx.

### Serotonergic nervous system

In this study, the serotonergic nervous system has been described for the first time within the genus *Monocelis* to allow comparisons between intact and regenerating animals*.* The names of major components of the serotonergic nervous system follow [41] for three monocelidid species. In *Monocelis* sp., we recognized the main, lateral, dorsal and ventral nerve cords described in [41] and several other minor longitudinal cords that seem to be part of the serotonergic nerve plexus. Compared with *Archilopsis* and *Promonotus*[41], the subepidermal serotonergic nerve plexus of *Monocelis* is particularly well developed (Figure 2E,G). The orthogonal pattern of the nervous system is well-known both in proseriates and other flatworms [36, 41].During regeneration, the severed main nerve cords, which are normally connected by a caudal loop, became reconnected after about 36 hours (Figure 6J). At 12 and 24 hours (Figure 6F, H), there were already some fine finger-like networks linking the main nerve cords. An explanation for this behaviour may be that the finger-like fibres were searching for signs of the other cord and once found, the main cords connected and the finger-like extensions disappeared.

In the macrostomorphan flatworm *Macrostomum lignano*, the loop of the main nerve cords in the tail plate is already reconnected 24 hours after amputation of the tail plate [23], close to our own findings for *Monocelis* sp. In the triclad *Schmidtea mediterranea*, where the caudal loop is less apparent and is formed like a wedge, reconnection of the main nerve cords is seen at least 15 days after amputation [42].

## Conclusions

Like many other flatworms with obligatory sexual reproduction, *Monocelis* sp. cannot regenerate a head after transverse amputation anterior to the pharynx. The regeneration blastema is a centre of proliferation, but the pharynx is regenerated anterior to the blastema in *Monocelis*. Only in triclads is the blastema devoid of proliferation, and this is probably a derived character. Like findings in other flatworms, there is no proliferation at the site of organ differentiation in *Monocelis*. The regeneration of the pharynx recapitulates organogenesis during embryonic development in that the pharynx primordium develops in a vertical orientation first and flips over horizontally at a later stage. This finding also suggests that a vertical pharynx is the plesiomorphic state in proseriates.

## Electronic supplementary material

Additional file 1: **Accession numbers of sequences of species used for the phylogenetic tree (see Additional file** 2 **).** The asterisk marks the species used for experiments in the present study. (XLS 10 KB)

Additional file 2: **Maximum likelihood phylogenetic tree reconstructed using 18S and 28S ribosomal RNA sequences of several proseriates (see Additional file** 1 **for accession numbers).** All *Monocelis* species are highlighted. The branch length scale indicates the number of substitutions per site. (JPEG 95 KB)

Additional file 3: **The diagonal actin fibres seem to be the outermost muscle layer.** Scale bar, 50 μm. (TIFF 2 MB)

Additional file 4: **A movie made from a confocal stack shows the diagonal actin fibres as the outermost muscle layer.** (AVI 4 MB)

Additional file 5: **A movie made from a confocal stack shows the premature pharynx of a posterior regenerate 48 hours post-amputation.** At this time point, the immature pharynx is still orientated in a vertical direction. (AVI 1 MB)

Additional file 6: **Incomplete pharynx regeneration of posterior regenerates 7 days post-amputation, revealed by phalloidin staining.** (TIFF 3 MB)

Additional file 7: **Anterior regenerates with phalloidin (green) and serotonin (red) staining.** White arrowheads in B and C show spots with weaker phalloidin staining. Scale bars are 100 μm. (TIFF 7 MB)

Additional file 8: **Phalloidin staining shows basket-shaped actin fibres, probably surrounding a gland cell.** Scale bars are 25 μm. (TIFF 613 KB)

Additional file 9: **Movie made from a confocal stack shows basket-shaped actin fibres.** (AVI 796 KB)

## Abbreviations

ASW: artificial sea water
BrdU: 5-bromo-2′-deoxyuridine
BSA-T: phosphate buffered saline with 0.1% Triton-X and 1% bovine serum albumin
F-actin: filamentous actin
PBS: phosphate buffered saline
PBS-T: phosphate buffered saline with 0.1% Triton-X
S-phase: synthesis phase
TRITC: tetramethylrhodamine isothiocyanate.
