# Supplementary figures and images for: Cellular dynamics during regeneration of the flatworm Monocelis sp. (Proseriata, Platyhelminthes)
Source: EvoDevo. 2014 Oct 23;5:37. doi: 10.1186/2041-9139-5-37 (PMC4407785; doi:10.1186/2041-9139-5-37)

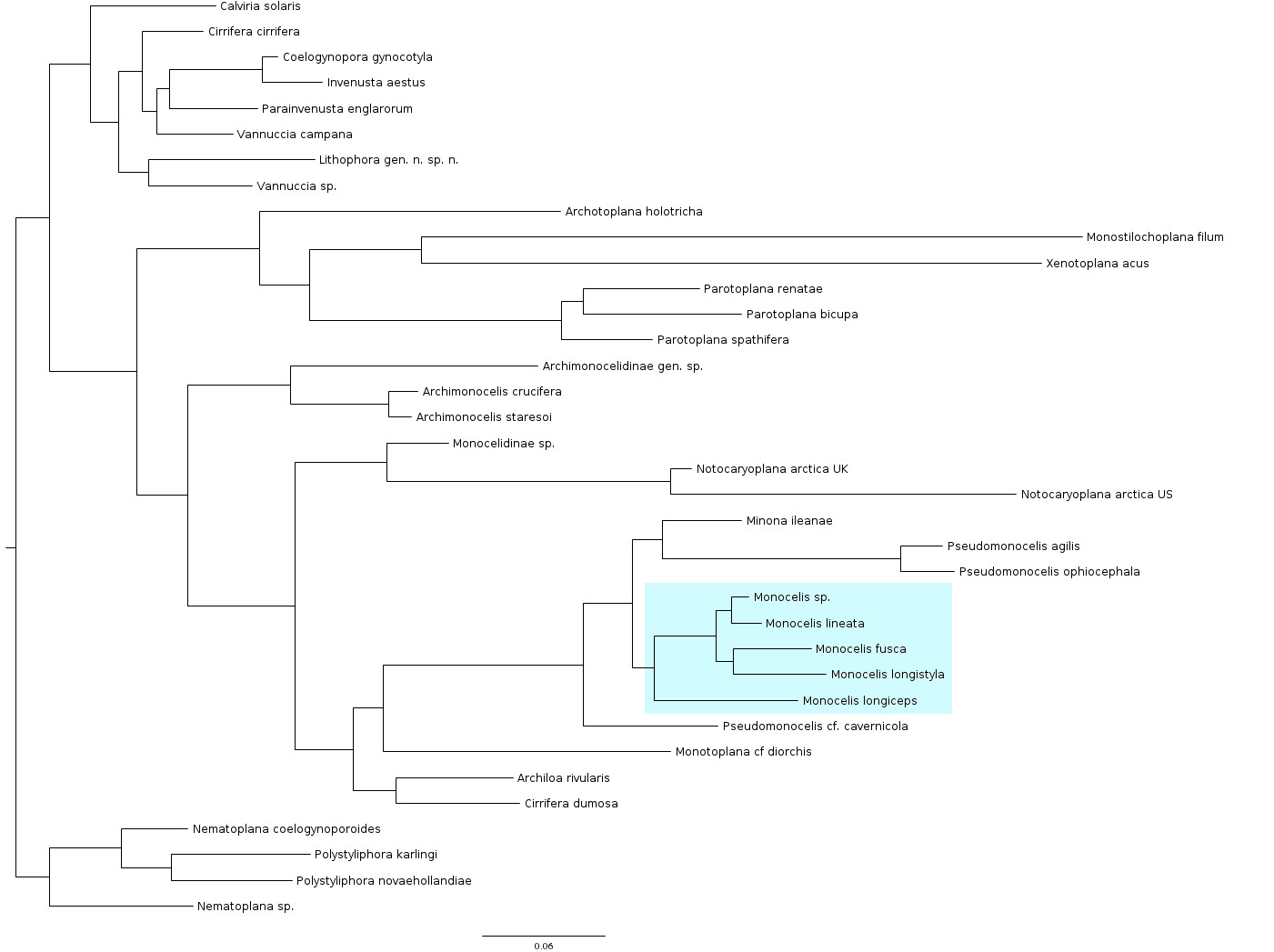

Supplement: Supplementary file 2 — Additional file 2: Maximum likelihood phylogenetic tree reconstructed using 18S and 28S ribosomal RNA sequences of several proseriates (see Additional file 1 for accession numbers). All Monocelis species are highlighted. The branch length scale indicates the number of substitutions per site. (JPEG 95 KB) [file 13227_2014_132_MOESM2_ESM.jpeg]

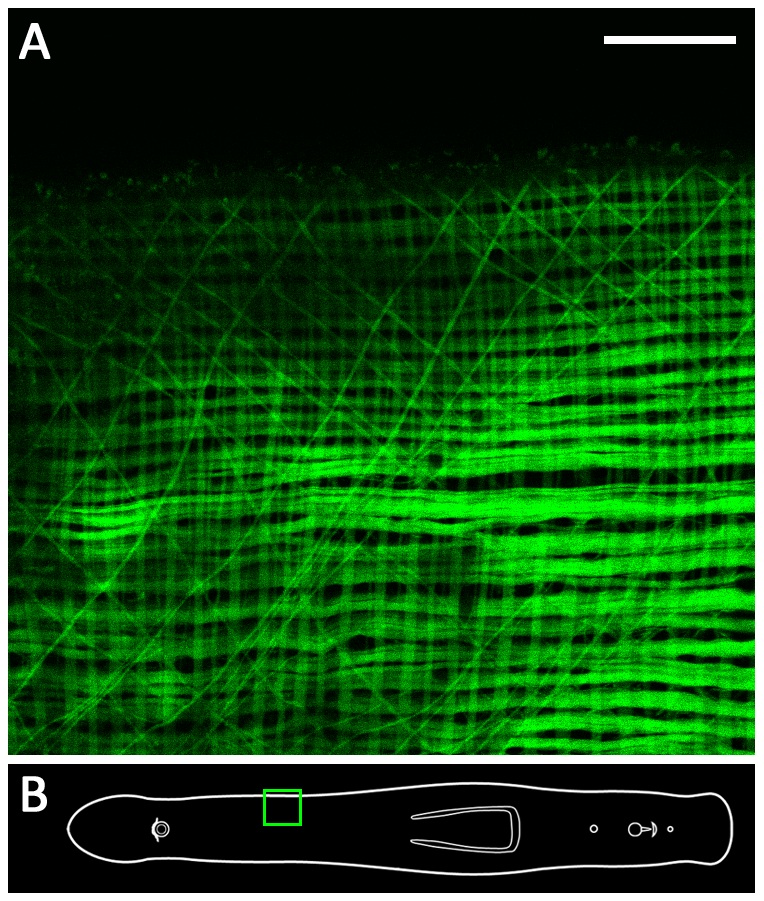

Supplement: Supplementary file 3 — Additional file 3: The diagonal actin fibres seem to be the outermost muscle layer. Scale bar, 50 μm. (TIFF 2 MB) [file 13227_2014_132_MOESM3_ESM.tiff]

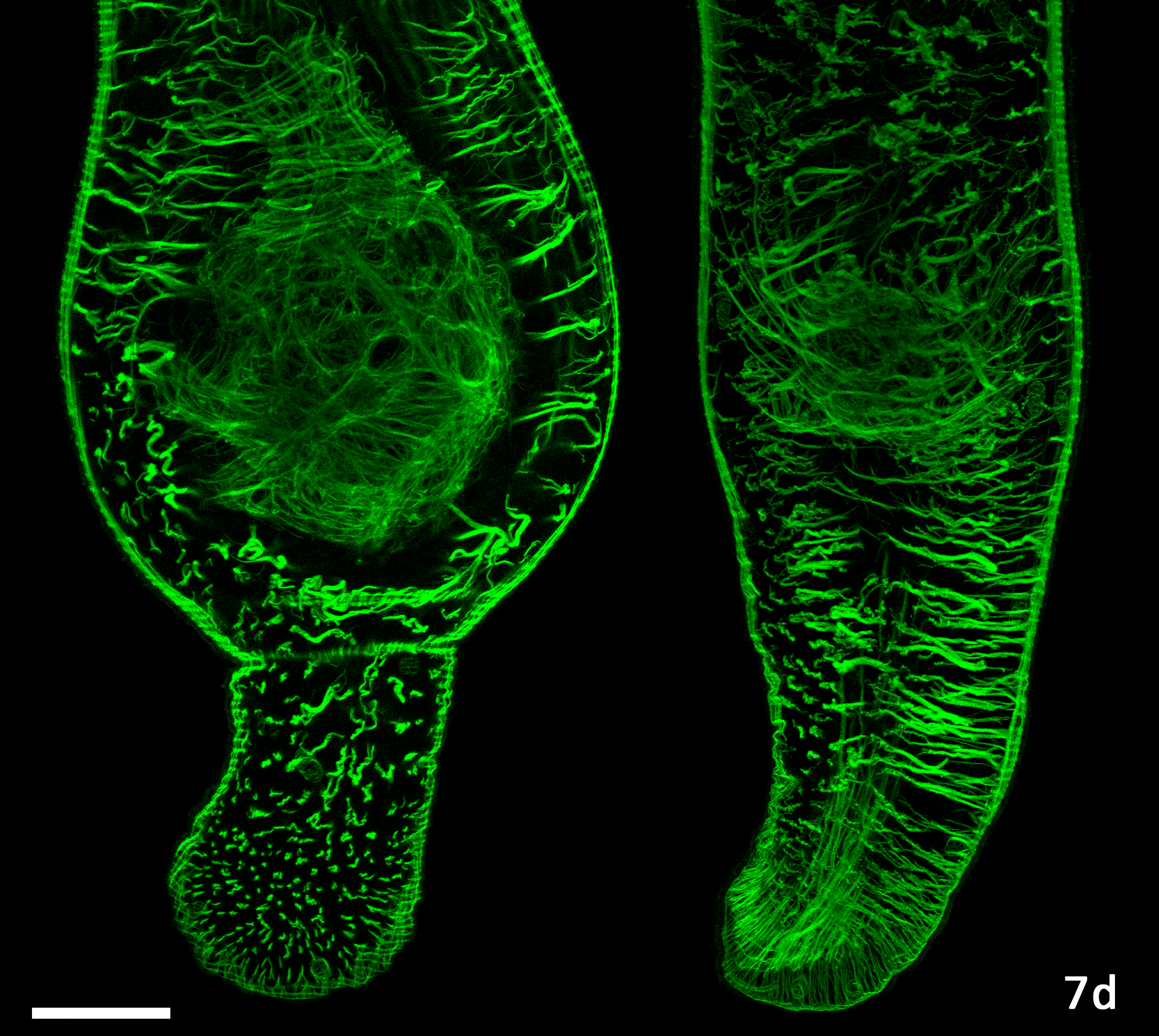

Supplement: Supplementary file 6 — Additional file 6: Incomplete pharynx regeneration of posterior regenerates 7 days post-amputation, revealed by phalloidin staining. (TIFF 3 MB) [file 13227_2014_132_MOESM6_ESM.tiff]

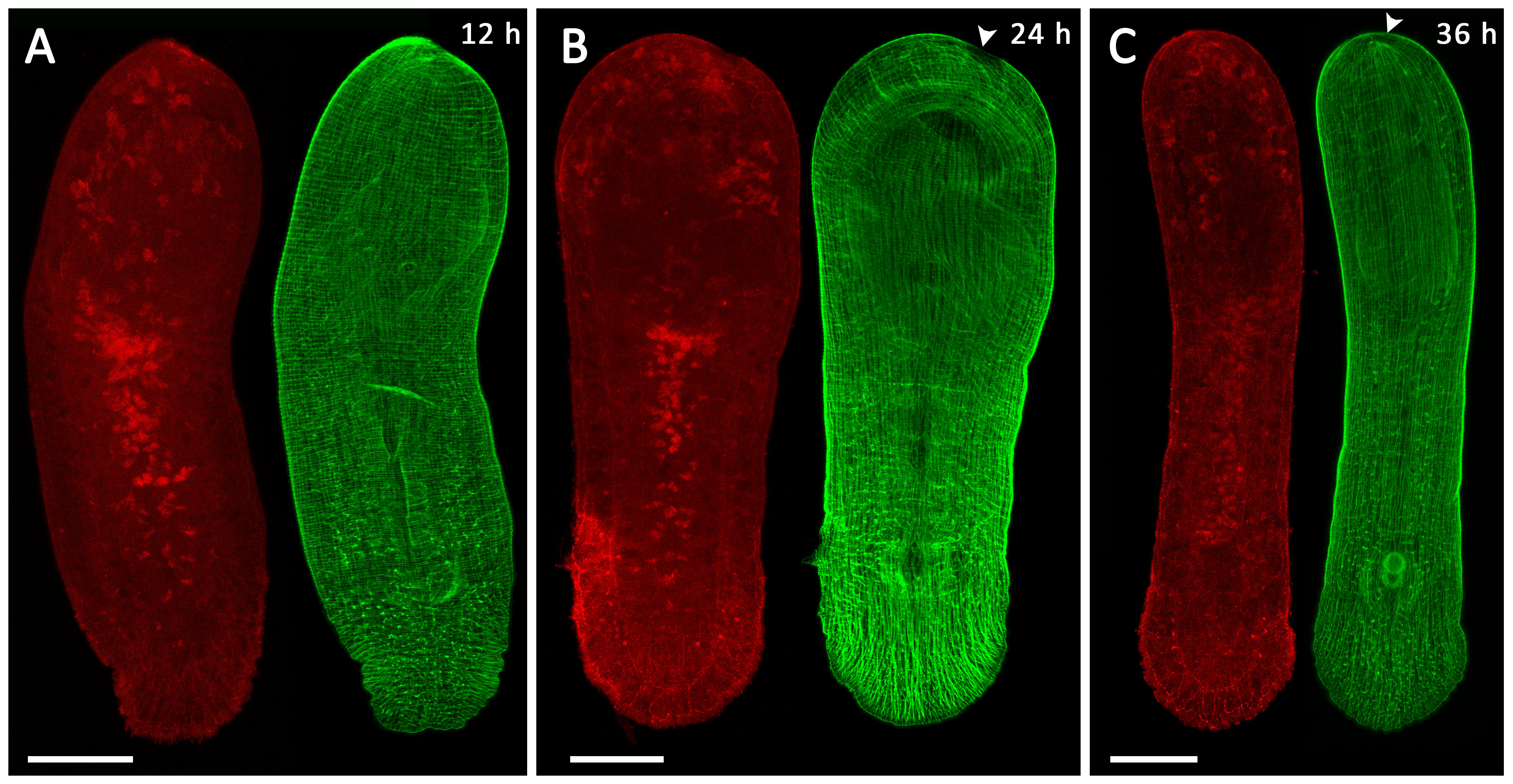

Supplement: Supplementary file 7 — Additional file 7: Anterior regenerates with phalloidin (green) and serotonin (red) staining. White arrowheads in B and C show spots with weaker phalloidin staining. Scale bars are 100 μm. (TIFF 7 MB) [file 13227_2014_132_MOESM7_ESM.tiff]

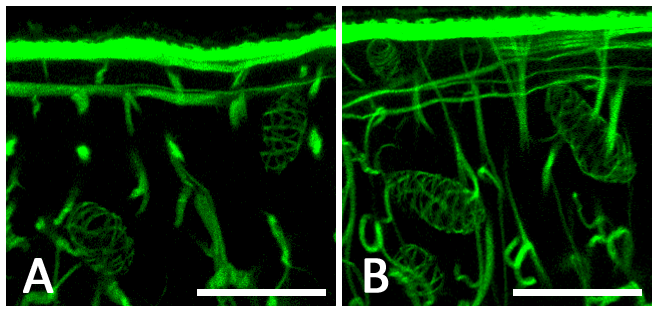

Supplement: Supplementary file 8 — Additional file 8: Phalloidin staining shows basket-shaped actin fibres, probably surrounding a gland cell. Scale bars are 25 μm. (TIFF 613 KB) [file 13227_2014_132_MOESM8_ESM.tiff]
